# Prognostic Value of Clinical Biochemistry-Based Indexes in Nasopharyngeal Carcinoma

**DOI:** 10.3389/fonc.2020.00146

**Published:** 2020-03-06

**Authors:** Xiaojiao Zeng, Guohong Liu, Yunbao Pan, Yirong Li

**Affiliations:** ^1^Department of Laboratory Medicine, Zhongnan Hospital of Wuhan University, Wuhan University, Wuhan, China; ^2^Department of Radiology, Zhongnan Hospital of Wuhan University, Wuhan University, Wuhan, China

**Keywords:** nasopharyngeal carcinoma, systemic inflammation score (SIS), albumin/globulin ratio (AGR), prognostic nutritional index (PNI), albumin-to-alkaline phosphatase ratio (AAPR)

## Abstract

Inflammation and nutritional status have significant effects on the prognosis of cancer patients. This study investigated the predictive value of clinical biochemistry-based indexes in nasopharyngeal carcinoma (NPC). This retrospective study included 559 NPC patients and 500 patients with chronic rhinitis. Continuous variables were measured by *t*-test. The area under curves (AUC) was used to determine the diagnostic and prognostic value for NPC. Kaplan-Meier methods and the log-rank test were used to analyze overall survival (OS) and disease-free survival (DFS) of the patients. Cox and logistic regression analysis were used to analyze the independent prognostic risk factors for survival and influencing factors of side effects after treatment, respectively. The study results revealed that most indexes of NPC and rhinitis were significantly different between the two groups. In the survival analysis, the systemic inflammation score (SIS), prognostic nutritional index (PNI), albumin/globulin ratio (AGR), albumin (ALB), urea nitrogen (BUN) and creatinine (CREA) had significant influence on the OS and DFS. AGR was the optimal prognostic indicator for NPC. Among these indexes, SIS, AGR, BUN and CERA were independent prognostic factors of OS, AGR and PNI were independent prognostic factors of DFS. Most indexes were risk factors of side effects occurred in radiotherapy. In conclusion, the clinical biochemistry-based indexes, are reliable and of low-cost, therefore, they can be used in predicting diagnosis, prognosis and treatment plans of NPC.

## Introduction

Nasopharyngeal carcinoma (NPC) is an epithelial cancer of the nasopharynx. Globally, it is a rare form of cancer, however, it is highly prevalent in Southeast Asia and Southern China (1). NPC staging is mainly based on the tumor-node-metastasis (TNM) staging system developed by the American Joint Cancer Committee staging (AJCC). This system is used for treatment selection, cancer control strategies and outcome prediction. Concurrent chemoradiotherapy has been regarded as the standard treatment for NPC because of the covert nature of the tumorigenic site and intrinsic sensitivity of radiotherapy (2, 3). Over the years, radiotherapy technology has evolved from two-dimensional radiotherapy (2DRT) to intensity-modulated radiotherapy (IMRT) and this has resulted in longer survival and milder toxicity in NPC patients (4). Chen et al. reported that NPC patients with IMRT had better failure-free survival and overall survival than patients with 2DRT, and the incidence of toxicity in the IMRT group, such as dry mouth, was lower than in the 2DRT group (5). Deutsch et al. also reported that IMRT had reduced the risk of long-term sequelae, such as xerostomia in patients with head and neck carcinoma (6). While there is still some acute toxicity when treated with radiotherapy, such as mucositis (7). Our study would explore the influencing factors of common side effects after radiotherapy for patients with NPC.

The influence of inflammation and nutrition status on the prognosis of patients has been reported in many cancer types (8, 9). The two-fold connection between cancer and inflammation had been reported in the early stage of cancers (10). The inflammatory reaction stimulated by cancer is beneficial to its growth, progression, and immunosuppression.

On the other hand, malnutrition has been associated with metabolic abnormalities and functionality changes besides dysphagia which is caused by head and neck cancer (11). In addition, anorexia can be induced by inflammatory mediators which directly or indirectly produced (12). The nutrition has a profound influence on leukocytes and proinflammatory carcinogenic action of the anticancer immune response (13). The nutritional status is closely related to the prognosis of cancer patients and the global incidence of cancer-associated malnutrition ranges from 30 to 85% (14).

The clinical biochemistry-based indexes, such as prognostic nutritional index (PNI), albumin-to-alkaline phosphatase ratio (AAPR), albumin/globulin ratio (AGR), albumin (ALB), serum urea nitrogen (BUN) and creatinine (CREA) have been considered as independent prognostic factors for many cancers (15–21). The systemic inflammation score (SIS), based on the serum albumin level and LMR, has shown a better predictive effect on the prognosis of cancer compared to the single index (22).

Low level of PNI, AAPR, AGR, and ALB had been reported to be associated with poor survival in NPC, however, the combined analysis has not been reported (16, 19, 23–26). Moreover, the studies on the effect of serum biochemical indexes on toxicity with radiotherapy and the prognostic value of SIS, BUN, and CREA for NPC have not yet been reported.

To investigate the effect of biochemistry-based indexes on the diagnosis and prognosis of NPC and side effects after radiotherapy. We retrospectively collected the clinical data of 559 NPC patients and 500 patients with rhinitis, and 255 NPC patients were further followed up. We explored the survival and side effects by cox and logistic regression analysis. Besides, we investigated the prognostic efficiency of these indexes by receiver operating characteristic curve. Owing to ignoring the functional status of NPC, patients with the same TNM staging may have different prognosis (27). These biological markers could supply the current evaluation system of TNM staging system to help predict the diagnosis and development for patients with NPC as inexpensive and straightforward prognostic predictors.

## Materials and Methods

### Patients

This restrospective study recruited 559 patients diagnosed with NPC at the Zhongnan Hospital of Wuhan University from January 2014 to November 2018. The patients comprised 421 males and 138 females with a median age of 51 (range 12–83 years). To verify the predictive value of the clinical biochemistry-based indexes for diagnosis and development of NPC, this retrospective study recruited new set of 500 patients diagnosed with rhinitis in the same period as normal controls. The patients comprised 312 males and 188 females with a median age of 46 (range 10–83 years). The seventh edition of the American Joint Committee on Cancer (AJCC) staging system was used for stage classification.

### Inclusion and Exclusion Criteria

Inclusion criteria in this study were: (1) patients with histopathological confirmation of NPC; (2) patients who exhibited normal renal, cardiac, and liver function to tolerate chemotherapy and radiotherapy; (3) patients with a complete record of hematological indicators and serum biochemical parameters. Exclusion criteria were as follows: (1) patients with other types of malignancy. Databased on these criteria, a total of 255 patients' data was retrieved and used for survival analysis.

### Hematological and Serum Biochemical Examination

The patients fasting whole blood was collected in an EDTA anticoagulant-treated tube and analyzed within 30 min. Routine peripheral blood cells, including lymphocytes and monocytes, were analyzed by Beckman Coulter DxH 800 automated blood analyzer and related reagents (Beckman, California, USA).

Routine serum biochemical parameters, including alanine aminotransferase (ALT), aspartate aminotransferase (AST), AST/ALT ratio, total bilirubin (TBIL), direct bilirubin (DBIL), unconjugated bilirubin (UBIL), total protein (TP), ALB, globulin (GLB), AGR, γ-glutamyl transpeptidase (GGT), alkaline phosphatase (ALP), total bile acid (TBA), glucose (GLU), BUN, CREA, uric acid (UA), and CO_2_, were measured by Beckman Coulter AU automatic biochemical analyzer and related reagents (Beckman, California, USA). The combined indexes, PNI, AAPR, LMR, and SIS, were defined as follows:

PNI: 10 × serum albumin level (g/dL) + 0.005 × total lymphocyte count (per mm^3^);
AAPR: Albumin/Alkaline Phosphatase;
LMR: lymphocytes/monocytes;
SIS: both Alb level < 40 g/l and LMR < 3.05 were assigned a score of 2; either Alb level ≥ 40 g/l or LMR ≥ 3.05 were assigned a score of 1; both Alb level ≥ 40 g/l and LMR ≥ 3.05 were assigned a score of 0 (28).

PNI, AAPR, and LMR were dichotomized at the median values. Other serological parameters were classified according to the limit values of the reference interval. The optimal cut-off values were as follows: PNI (45.58), AAPR (0.63), LMR (3.05), SIS (1), ALB (40), AGR (1.5), BUN (7.6), CREA (104).

### Follow Up

The primary endpoint and secondary endpoint were the overall survival (OS) and disease-free survival (DFS), respectively. Patients diagnosed as NPC were followed up primarily by telephone and periodic review at the hospital. Out of the 559 patients participating in the study 255 patients were followed up successfully. OS was defined as the period from the initial diagnosis to the last follow-up or death regardless of whether it was NPC related or not. The median follow-up time among the 255 patients was 33.5 months, ranging from 2.1 to 151.2 months. DFS was defined as the period from the initial diagnosis to recurrence or metastasis. All the follow-ups ended in February 2019.

### Statistical Analysis

Statistical analyses were performed using IBM SPSS version 22.0 software (SPSS, Chicago, IL). One-way ANOVA and LSD tests were used to test for the serological parameters among multiple subgroups. Continuous variables were measured by the *t*-test and plotted by GraphPad Prism V7.0 software. Pearson correlation was used to determine the relationship between SIS and clinical indexes. Kaplan-Meier methods and the log-rank test were used to estimate the OS and DFS. Univariate and multivariate Cox proportional hazards regression model were used to determine the independent prognostic risk factors for survival. The univariate and multivariate logistic regression analysis were used to analyze the influencing factors for side effects during treatment. The receiver operating characteristic (ROC) curve was used to evaluate the predictive and prognostic accuracy of clinical biochemistry-based indexes. A *p*-value < 0.05 is considered as statistically significant.

## Results

### Baseline Characteristics of NPC and Rhinitis Patients

**Figure 1:**
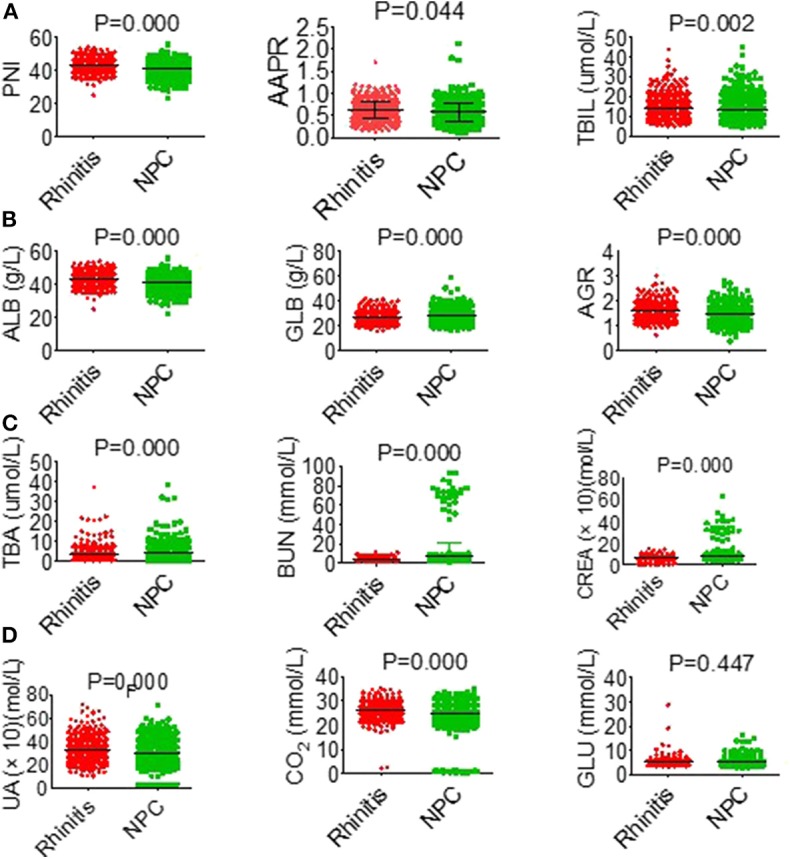
General characteristics of hematological parameters between NPC and rhinitis patients. **(A)** PNI (left), AAPR (middle), TBIL (right). **(B)** ALB (left), GLB (middle), AGR (right). **(C)** TBA (left), BUN (middle), CREA (right). **(D)** UA (left), CO_2_ (middle), GLU (right).

**Figure 2:**
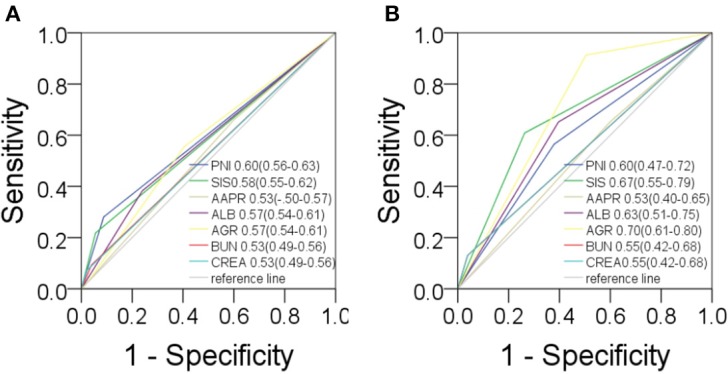
Predictive values in NPC. For diagnosis **(A)** and prognosis **(B)** of PNI, SIS, AAPR, ALB, AGR, BUN, CREA.

Based on the study results, there was a high prevalence of NPC among in men and young people (Table S1). Serological parameters between NPC and rhinitis patients were shown in Figure 1. Most of the parameters between the two cohorts, such as PNI, AAPR, AGR, ALB, BUN, and CREA were significantly different. ROC curve was used to investigated the diagnostic efficacy of indexes (Figure 2A). The predictive value of the PNI (0.60, 0.56–0.63) for NPC diagnosis was superior to that of the AAPR, SIS, ALB, AGR, BUN, and CREA.

### The Association Between Clinical Indexes and Serological Parameters in NPC Patients

**Figure 3:**
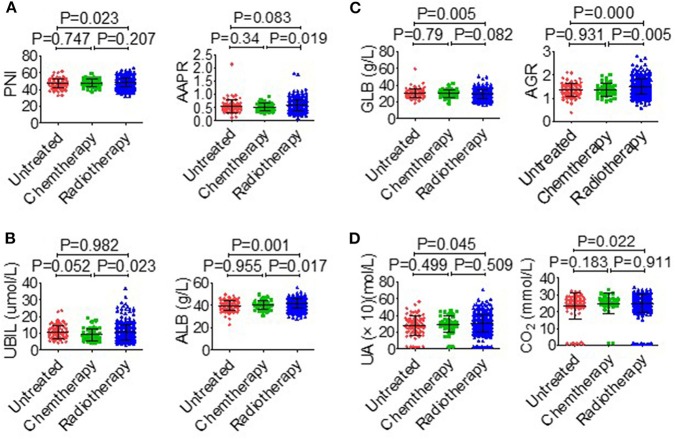
Effects of therapy on serological parameters. **(A)** PNI (left), AAPR (right). **(B)** UBIL (left), ALB (right). **(C)** GLB (left), AGR (right). **(D)** UA (left), CO_2_ (right).

**Figure 4:**
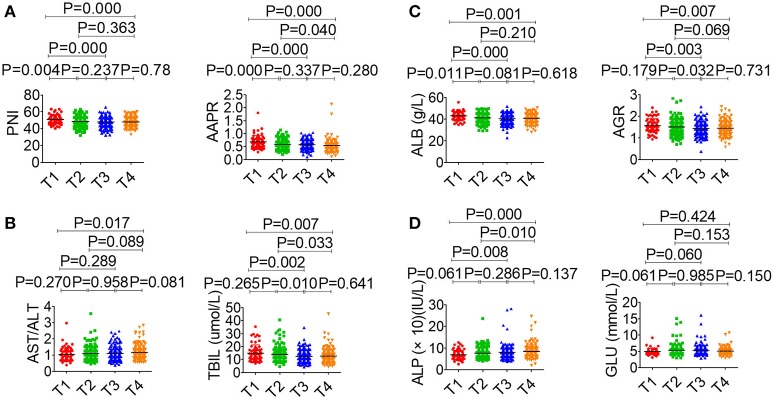
Effects of T stage on serological parameter. **(A)** PNI (left), AAPR (right). **(B)** AST/ALT (left), TBIL (right). **(C)** ALB (left), AGR (right). **(D)** ALP (left), GLU (right).

**Figure 5:**
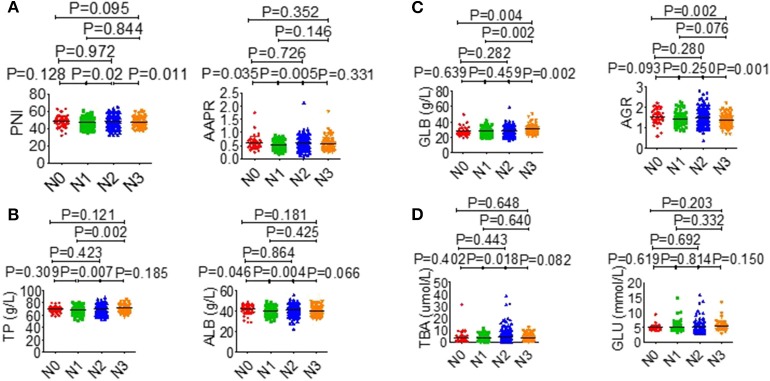
Effects of N stage on serological parameter. **(A)** PNI (left), AAPR (right). **(B)** TP (left), ALB (right). **(C)** GLB (left), AGR (right). **(D)** TBA (left), GLU (right).

The association between serological parameters and clinical characteristics in 559 NPC patients were shown in Table 1. The serological parameters in a different circumstance, including therapy, TNM staging system, were displayed in Figures 3–5. Significant differences for the serological parameters are diverse in sex, age, therapy, and different TNM stage groups, such as the differences of PNI, AAPR, AGR, and ALB in sex, age, therapy, T, N, and M stage groups. Multiple comparative analyses showed that there were significant differences in the clinical biochemistry-based indexes (such as PNI and AAPR), as shown between untreated and chemotherapy or radiotherapy, T stage of 1 or 2 and T stage of 3 or 4 and N stage of 0 or 1 and N stage of 2 or 3 (Figures 3–5).

**Table 1:** General characteristics of serological parameters of 559 included subjects.

**Parameters**	**Sex**	**x ± s**	***p***	**Age**	**x ± s**	***p***	M* stage	**x ± s**	***p***
PNI	M	48.58 ± 5.66	0.69	<60	49.17 ± 5.44	0.00	M0	48.89 ± 5.34	0.00
	F	48.36 ± 5.05		≥60	46.53 ± 5.27		M1	45.81 ± 6.01	
AAPR	M	0.55 ± 0.18	0.00	<60	0.59 ± 0.19	0.00	M0	0.59 ± 0.20	0.00
	F	0.65 ± 0.24		≥60	0.53 ± 0.22		M1	0.46 ± 0.15	
ALT	M	30.35 ± 71.23	0.08	<60	29.54 ± 71.29	0.22	M0	28.08 ± 66.19	0.69
	F	19.56 ± 12.25		≥60	21.98 ± 11.62		M1	24.79 ± 13.64	
AST	M	27.25 ± 43.5	0.20	<60	26.8 ± 43.51	0.43	M0	26.06 ± 40.48	0.99
	F	22.46 ± 10.99		≥60	23.81 ± 10.73		M1	26.1 ± 11.64	
AST/ALT	M	1.06 ± 0.41	0.00	<60	1.09 ± 0.43	0.02	M0	1.10 ± 0.40	0.06
	F	1.27 ± 0.41		≥60	1.19 ± 0.39		M1	1.22 ± 0.51	
TBIL	M	13.36 ± 5.27	0.54	<60	13.17 ± 5.49	0.40	M0	13.45 ± 5.44	0.04
	F	13.03 ± 5.56		≥60	13.61 ± 4.85		M1	12.02 ± 4.35	
DBIL	M	2.65 ± 1.49	0.04	<60	2.51 ± 1.51	0.09	M0	2.58 ± 1.49	0.83
	F	2.35 ± 1.36		≥60	2.76 ± 1.32		M1	2.54 ± 1.24	
UBIL	M	10.71 ± 4.24	0.95	<60	10.65 ± 4.46	0.64	M0	10.87 ± 4.41	0.01
	F	10.68 ± 4.62		≥60	10.85 ± 3.93		M1	9.49 ± 3.50	
TP	M	69.63 ± 6.11	0.00	<60	70.69 ± 6.09	0.00	M0	70.00 ± 5.96	0.40
	F	71.44 ± 5.85		≥60	68.20 ± 5.75		M1	70.66 ± 6.99	
ALB	M	41.1 ± 4.47	0.48	<60	41.69 ± 4.29	0.00	M0	41.37 ± 4.23	0.00
	F	41.38 ± 3.91		≥60	39.55 ± 4.10		M1	4.85	
GLB	M	28.53 ± 5.21	0.00	<60	29.00 ± 5.19	0.51	M0	28.62 ± 5.14	0.00
	F	30.07 ± 5.16		≥60	28.65 ± 5.38		M1	31.02 ± 5.52	
AGR	M	1.49 ± 0.33	0.03	<60	1.49 ± 0.32	0.07	M0	1.49 ± 0.32	0.00
	F	1.42 ± 0.3		≥60	1.43 ± 0.31		M1	1.32 ± 0.28	
GGT	M	32.62 ± 34.6	0.01	<60	30.98 ± 38.28	0.47	M0	29.24 ± 32.49	0.15
	F	23.48 ± 36.65		≥60	28.46 ± 23.93		M1	38.55 ± 51.07	
ALP	M	81.66 ± 28.43	0.00	<60	78.74 ± 30.54	0.47	M0	77.20 ± 27.1	0.00
	F	71.83 ± 27.19		≥60	80.76 ± 20.61		M1	94.19 ± 33.32	
TBA	M	4.3 ± 3.39	0.88	<60	4.29 ± 4.04	0.92	M0	4.26 ± 3.84	0.71
	F	4.23 ± 5.32		≥60	4.25 ± 3.68		M1	4.45 ± 4.72	
GLU	M	5.28 ± 1.4	0.07	<60	5.19 ± 1.37	0.40	M0	5.22 ± 1.38	0.78
	F	5.04 ± 1.11		≥60	5.30 ± 1.21		M1	5.17 ± 0.89	
BUN	M	8.5 ± 14.89	0.01	<60	7.35 ± 12.82	0.14	M0	7.58 ± 13.02	0.35
	F	5.78 ± 8.03		≥60	9.31 ± 15.61		M1	9.61 ± 17.08	
CREA	M	88.32 ± 67.33	0.00	<60	78.45 ± 56.31	0.07	M0	80.44 ± 59.84	0.31
	F	61.24 ± 34.23		≥60	91.43 ± 76.12		M1	90.36 ± 75.51	
UA	M	318.12 ± 106.39	0.00	<60	296.88 ± 104.06	0.44	M0	302.30 ± 103.79	0.03
	F	240.04 ± 71.99		≥60	304.90 ± 106.12		M1	273.48 ± 107.23	
CO2	M	24.59 ± 6.25	0.38	<60	24.84 ± 5.83	0.39	M0	24.78 ± 5.84	0.45
	F	25.1 ± 5.32		≥60	24.33 ± 6.60		M1	24.19 ± 7.31	

M*, metastasis of tumor, nodes, metastasis staging system; M, Male; F, Female; PNI, prognostic nutritional index; AAPR, albumin-to-alkaline phosphatase ratio; ALT, alanine aminotransferase; AST, aspartate aminotransferase; TBIL, total bilirubin; DBIL, direct bilirubin; UBIL, unconjugated bilirubin; TP, total protein; ALB, albumin; GLB, globulin; AGR, albumin/globulin; GGT, γ-glutamyl transpeptidase; ALP, alkaline phosphatase; TBA, total bile acid; GLU, glucose; BUN, urea nitrogen; CREA, creatinine; UA, uric acid.

### Influence of Clinical Indexes and Hemograms on Side Effects

In this study, 500 out of 559 patients received radiotherapy (Table S2). Some of the common side effects of treatment were bone marrow suppression, radiodermatitis, radiation stomatitis, skin pigmentation after radiotherapy, dysphagia, gastrointestinal reaction, and innutrition. Some of the patients experienced side effects, such as bacterial infection, secondary anemia, hypoproteinemia, post-radiotherapy molt, electrolyte disturbances, secondary thrombocytopenia, abnormal liver function, and agranulocytosis. This study also focused on the factors affecting the treatment side effects. Results analyzed by univariate and multivariate logistic regression analysis were shown in Figure S1. In multivariate logistic regression analysis, AJCC staging system, PNI and SIS were found to be independent risk factors for the radiodermatitis (Figure S1A), whereas PNI, AST, ALB, and BUN were independent risk factors for radiation stomatitis (Figure S1B). Besides, AST was the independent risk factor for skin pigmentation after radiotherapy (Figure S1C). The independent risk factors for dysphagia included PNI and ALB (Figure S1D), and the independent risk factors for the gastrointestinal reaction included PNI and AGR (Figure S1E). The independent risk factors for innutrition included AAPR, ALB, and AGR (Figure S1F). In univariate logistic regression analysis, ALB had impact on the arrest of suppression (Figure S1G). Results of most indicators on side effects in univariate analysis were consistent with that in multivariate analysis except arrest of bone marrow and dysphagia.

### Clinical Characteristics of Patients With NPC in Survival Analysis

Survival analysis included 255 patients (Table S2), of these 202 were male while 53 were female patients with NPC. The patients' median age was 51 years (range from 12–78 years). The correlations between SIS and clinical characteristics are summarized in Table 2. High-score SIS were correlated with low-score PNI, low-score AAPR and low-score AGR.

**Table 2:** Clinical characteristics of patients with NPC.

**Variables**	***n***	**SIS**		***P*-value**
	**255**	**0–1 (*n* = 180)**	**2 (*n* = 75)**	
Therapy				0.64
Untreated	8	5 (2.8%)	3 (4.0%)	
Chemotherapy alone	15	12 (6.7%)	3 (4.0%)	
Chem-radiotherapy	209	145 (80.5%)	64 (85.3%)	
Radiotherapy alone	23	18 (10.0%)	5 (6.7%)	
Sex				0.23
M	202	139 (77.2%)	63 (84.0%)	
F	53	41 (22.8%)	12 (16.0%)	
Age				0.07
<60	193	142 (78.9%)	51 (68.0%)	
≥60	62	38 (21.1%)	24 (32.0%)	
Stage				0.75
I-III	38	26 (14.4%)	12 (16.0%)	
IV	217	154 (85.6%)	63 (84.0%)	
Histology (WHO)				0.43
Keratinizing^*^	6	5 (2.8%)	1 (1.3%)	
Non-Keratinizing^#^	243	172 (95.5%)	71 (94.7%)	
Unknown	6	3 (1.7%)	3 (4%)	
PNI				0.00
≥45.58	154	147 (81.7%)	7 (9.3%)	
<45.58	101	33 (18.3%)	68 (90.7%)	
AAPR				0.00
≥0.63	101	83 (46.1%)	18 (24.0%)	
<0.63	154	97 (53.9%)	57 (76.0%)	
AGR				0.00
1.5-2.5	117	97 (53.9%)	20 (26.7%)	
<1.5	138	83 (46.1%)	55 (73.3%)	
BUN				0.76
<7.6	243	172 (95.6%)	71 (94.7%)	
≥7.6	12	8 (4.4%)	4 (5.3%)	
CREA				0.73
<104	243	171 (95.0%)	72 (96.0%)	
≥104	12	9 (5.0%)	3 (4.0%)	

* *Keratinizing squamous cell carcinoma*;

# *Non-Keratinizing carcinoma*.

### Associations of Clinical Biochemistry-Based Indexes With Survival

**Figure 6:**
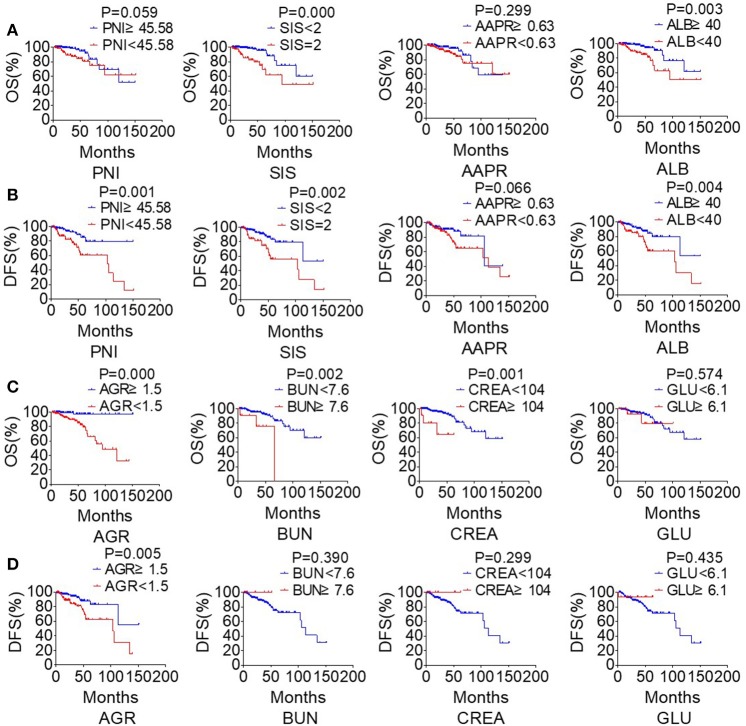
The clinical biochemistry-based indexes predict survival in NPC. Estimated overall survival (OS) **(A)** and disease-free survival (DFS) **(B)** curves for PNI, SIS, AAPR, ALB. Estimated overall survival (OS) **(C)** and disease-free survival (DFS) **(D)** curves for AGR, BUN, CREA, GLU.

The study took OS and DFS as the primary and secondary outcome, respectively. Non-metastatic patients were also included in the DFS analysis. The median follow-up time for OS was 33.5 months (range from 2.1 to 151.2 months), and 28.4 months (range from 1 to 151.2 months) for DFS. Based on the cut-off values, patients were subdivided into low-score and high-score groups of various indicators. By Kaplan-Meier analysis and the log-rank test, hematological indexes, such as AGR and ALB, which in low-score groups were significantly associated with worse OS in NPC patients, while high-score groups of SIS, BUN, and CREA were significantly associated with worse OS. In addition, NPC patients with low-score groups of indexes, including PNI, AGR and ALB, and patients with high-score group of SIS were significantly associated with worse DFS, while AAPR and GLU had little effect on OS and DFS (Figure 6). In univariate Cox regression analysis, OS was significantly affected by SIS, AGR, BUN, and CREA (Table 3), and DFS was affected by PNI and AGR (Table 4). While in multivariate Cox regression analysis, for OS, SIS (*P* = 0.01; HR = 3.17; 95% CI:1.30–7.73), AGR (*P* = 0.00; HR = 11.75; 95% CI:2.40–57.41), BUN (*P* = 0.02; HR = 4.91; 95% CI:1.33–18.14), and CREA (*P* = 0.00; HR = 11.61; 95%CI:2.80–48.22) were independent prognostic risk factors (Table 3). And for DFS, PNI (*P* = 0.01; HR = 2.64; 95% CI:1.29–5.41), and AGR (*P* = 0.03; HR = 2.27; 95% CI:1.07–4.81) were independent prognostic risk factors (Table 4). Moreover, the predictive value of the AGR on the prognosis of NPC was superior to that of other indexes (Figure 2B).

**Table 3:** Univariate and multivariate Cox proportional hazards regression analysis for OS.

**Variables**	**Univariate**			**Multivariate**		
	**HR**	**95CI**	***P*-values**	**HR**	**95CI**	***p*-values**
Stage	1.2	0.28–5.16	0.81			
I-III	Ref.					
IV						
Histology (WHO)			0.98			
Keratinizing	Ref.					
Non-Keratinizing	62767.17	0−2.12E+275	0.97			
Unknown	75741.20	0−2.57E+275	0.97			
PNI	2.18	0.95–4.97	0.07			
≥45.58	Ref.					
<45.58						
SIS	4.17	1.80–9.65	0.00	3.17	1.30–7.73	0.01
<2	Ref.			Ref.		
2						
AAPR	1.57	0.67–3.72	0.30			
≥0.63	Ref.					
<0.63						
ALB	3.42	1.45–8.08	0.01	0.34	0.03–3.73	0.38
≥40.00	Ref.			Ref.		
<40.00						
AGR	10.25	2.40–43.77	0.00	11.75	2.40–57.41	0.00
≥1.5	Ref.			Ref.		
<1.5						
BUN	5.49	1.59–18.93	0.01	4.91	1.33–18.14	0.02
<7.6	Ref.			Ref.		
≥7.6						
CREA	6.52	1.85–23.00	0.00	11.61	2.80–48.22	0.00
<104	Ref.			Ref.		
≥104						

**Table 4:** Univariate and multivariate Cox proportional hazards regression analysis for DFS.

**Variables**	**Univariate**			**Multivariate**		
	**HR**	**95CI**	***P*-values**	**HR**	**95CI**	***p*-values**
Stage	1.55	0.47–5.11	0.47			
I-III	Ref.					
IV						
Histology (WHO)			0.46			
Keratinizing	Ref.					
Non-Keratinizing	23415.15	0−2.72E+122	0.94			
Unknown	51937.25	6.06E+122	0.94			
PNI	3.14	1.55–6.36	0.00	2.64	1.29–5.41	0.01
≥45.58	Ref.			Ref.		
<45.58						
SIS	2.83	1.44–5.54	0.00	1.65	0.48–5.73	0.43
<2	Ref.			Ref.		
2						
AAPR	1.98	0.94–4.14	0.07			
≥0.63	Ref.					
<0.63						
ALB	2.60	1.32−5.14	0.01	0.66	0.15−2.86	0.58
≥40.00	Ref.			Ref.		
<40.00						
AGR	2.78	1.33–5.79	0.01	2.27	1.07–4.81	0.03
≥1.5	Ref.			Ref.		
<1.5						
BUN	0.05	0.00–1638.68	0.57			
<7.6	Ref.					
≥7.6						
CREA	0.05	0.00–288.50	0.49			
<104	Ref.					
≥104						

## Discussion

This study results revealed that clinical biochemistry-based indexes, such as PNI, SIS, AGR, ALB, BUN, and CREA were valuable for the prediction of diagnosis and prognosis. These influenced common side effects during radiotherapy and chemotherapy. All the indexes were statistically significant predictors of survival of NPC patients. Among the indexes, SIS, AGR, BUN, and CREA were independent prognostic factors for OS. The death risk in patients with low AGR was 11.75 times higher than that in the high-score group. The risk of death in the high-score groups of the SIS, BUN, and CREA were 3.17, 4.91, and 11.61 times higher than those in the low-score groups of SIS, BUN, and CREA, respectively. Besides, PNI and AGR were independent prognostic factors for DFS. The risks of death in patients in the low-score group of the PNI and AGR were 2.64 and 2.27 times higher than those in the high-score group of the PNI and AGR, respectively.

Tumor microenvironment contains cancer cells, non-cancer cells, and the cancer surrounding matrix. The metabolism of cancer cells involves the development of inflammatory processes in the tumor microenvironment. Inflammation plays an essential role in the initiation and development of malignancies. Cancer-related immune and inflammatory response is a complex interaction (17). Inflammatory cells infiltrate significantly correlate with the outcome of solid cancers in the tumor microenvironment. Such as the macrophages in tissue evolve from the monocytes in the blood, which can promote angiogenesis, lymphangiogenesis, immunosuppression, and support tumor growth, invasion, and metastases (29). Conversely, lymphocytes have anti-cancer property and are associated with better prognosis in various solid tumors (17, 30). ALB is an acute-phase protein regarded as a marker of systemic inflammation, which enabling ALB closely related to the occurrence and development of cancer. ALB is positively correlated with the nutritional status of the patients. Patients undergoing cancer-related treatment often suffer from the gastrointestinal reaction, such as nausea and vomiting, and this contributes malnutrition in patients (31). This study suggested that NPC patients with low-level serum ALB had a short OS and DFS.

Cancer-related inflammation and malnutrition negatively affect cancer outcome. Liu et al. reported that inflammatory and nutritional markers were independent predictive indexes for the survival of gastric cancer patients (14). Combined indexes of inflammation and nutrition, such as PNI (18, 32), SIS (22, 33), and AGR (16, 34) have been proved to be related to survival in cancers. Consistently, our study suggested that low level of PNI and AGR and high level of SIS had poor OS and DFS in NPC patients. In this study, PNI that combined lymphocyte and ALB was the independent prognostic indicator for DFS, while SIS that combined LMR and ALB was the independent prognostic indicator for OS. AGR is a combined biomarker of albumin and globulin. Albumin reflects both the nutritional and inflammation status in human, while globulin reflects the status of the immune inflammation. Recent studies have suggested that AGR can predict survival in various cancers, such as esophageal squamous cell carcinoma, gastric cancer. In this study, AGR was the independent prognostic indicator for OS and DFS. Besides, ALP is a biomarker related to the bone metastasis in cancer. Kim et al. indicated that AAPR, a combination of ALB and ALP, can predict the prognosis for NPC patients (19). Although our result was inconsistent with the conclusion, AAPR had significant difference in the TNM stage of NPC patients. Moreover, high-score SIS were significantly correlated with low-score PNI, low-score AGR, and low-score AAPR.

To meet the basic survival and biosynthetic demands, the metabolism of proliferating cells, such as the cancer cells and stimulated immune cells, differ from those of static tissues (35). The BUN and CREA reflected the renal function, and high-level CREA was associated with vascular damage (36). This mechanism would contribute to the poor OS in NPC patients with high-level BUN and CREA in our study. Besides, our study suggested that BUN and CREA were the independent prognostic indicator for OS. There was also a positive correlation between high-score SIS and high-score BUN and high-score CREA.

Cancer cells show greater metabolic autonomy supporting their growth and proliferation compared to non-transformed cells. Also, metabolism has a shift from oxidative to fermentative metabolism (the Warburg effect) (37). In this study, there was no significant difference in GLU level between chronic rhinitis and NPC. Therefore, GLU level was not significant in prognosis and survival of NPC patients.

Radiotherapy is the main treatment modality in NPC with or without synchronous chemotherapy. The loco-regional control rate has increased, but with no clear improvement in OS and DFS of NPC (24). Sun et al. (38) reported that the combination of induction chemotherapy and concurrent chemoradiotherapy significantly improved the survival of locoregionally advanced NPC patients. Cisplatin combined with other drugs is often used for chemotherapy or chem-radiotherapy in head and neck cancer. In the current study, the patients were mainly treated with cisplatin-based chemotherapy regimens. Accompanied by, there were some acute toxicity during treatment (7). This study focused on seven common side effects during radiotherapy: bone marrow suppression, radiodermatitis, radiation stomatitis, skin pigmentation after radiotherapy, dysphagia, gastrointestinal reaction, innutrition. Among all side effects, bone marrow suppression was the highest. Moreover, many serological markers acted as risk factors for side effects, such as PNI, SIS, AGR, ALB, AAPR, and BUN.

This study had limitations for example, most NPC patients fail to follow up, and there were incomplete histopathological classification and grades. Besides, EBV is closely associated with NPC and circulating EBV DNA in plasma is useful for screening for early asymptomatic NPC. While the items of EB virus load and correlated antibody were regarded as regular tests for patients with NPC from the second half of 2017 in Zhongnan Hospital, while our retrospective study started in 2014. The correlation between immunological indicators and EBV is not analyzed.

In conclusion, the clinical biochemistry-based indexes, such as SIS, which can be used to predict diagnosis and prognosis of NPC. These indicators can help to estimate the quality of life of patients after treatment and this would be beneficial in the prevention of severe side effects.

## Data Availability Statement

The datasets generated for this study are available on request to the corresponding author.

## Ethics Statement

The studies involving human participants were reviewed and approved by Zhongnan Hospital of Wuhan University Ethics and Scientific Committee. Written informed consent to participate in this study was provided by the participants' legal guardian/next of kin.

## Author Contributions

YP conceived and designed the manuscript. XZ, GL, and YP acquired, analyzed, and interpreted the data and wrote and reviewed the manuscript. YL supervised the study.

### Conflict of Interest

The authors declare that the research was conducted in the absence of any commercial or financial relationships that could be construed as a potential conflict of interest.

## Supplementary Material

Figure S1 Univariate and multivariate logistic regression analysis of clinical parameters and hemograms on the side effects in NPC patients. **(A)** Radiodermatitis. **(B)** Radiation stomatitis. **(C)** Skin pigmentation after radiotherapy. **(D)** Dysphagia. **(E)** Gastrointestinal reaction. **(F)** Innutrition. **(G)** Arrest of bone marrow.

Table S1 General character of NPC and Rhinitis cohort.

Table S2 Therapeutic regimens of NPC patients.
