# Supplementary figures and images for: Prognostic Value of Clinical Biochemistry-Based Indexes in Nasopharyngeal Carcinoma
Source: Front Oncol. 2020 Mar 6;10:146. doi: 10.3389/fonc.2020.00146 (PMC7068812; doi:10.3389/fonc.2020.00146)

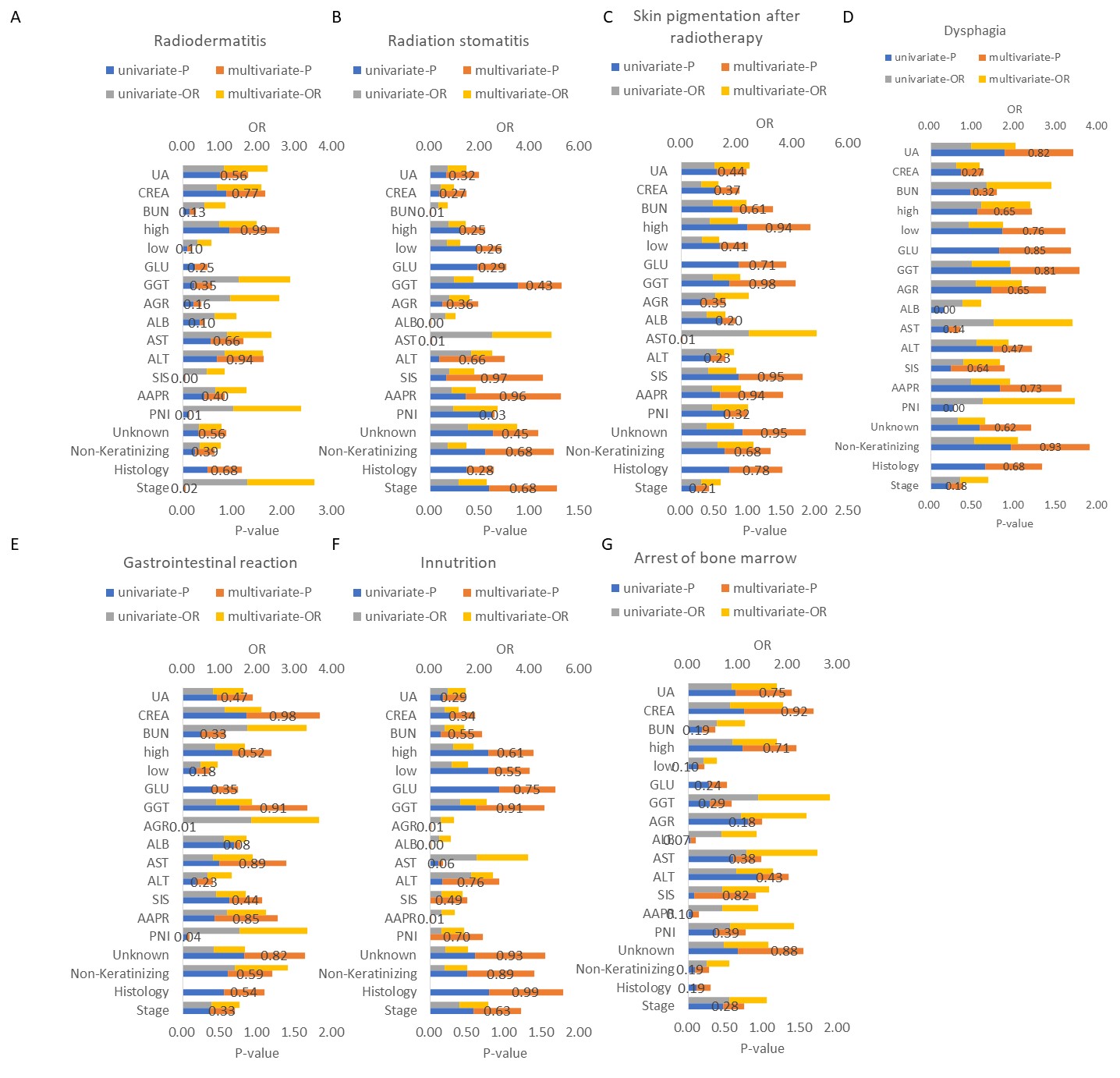

Supplement: Figure S1 — Univariate and multivariate logistic regression analysis of clinical parameters and hemograms on the side effects in NPC patients. (A) Radiodermatitis. (B) Radiation stomatitis. (C) Skin pigmentation after radiotherapy. (D) Dysphagia. (E) Gastrointestinal reaction. (F) Innutrition. (G) Arrest of bone marrow. [file Image_1.JPEG]
