# Supplementary material for: Prognostic Value of Clinical Biochemistry-Based Indexes in Nasopharyngeal Carcinoma
Source: Front Oncol. 2020 Mar 6;10:146. doi: 10.3389/fonc.2020.00146 (PMC7068812; doi:10.3389/fonc.2020.00146)
Supplement: Table S1 — General character of NPC and Rhinitis cohort. [file Table_1.docx]

Table S1 General character of NPC and Rhinitis cohort

| Variables | NPC (%) | Rhinitis (%) |
| --- | --- | --- |
|  |  |  |
| Sex |  |  |
| M | 421(75.3%) | 312(62.4%) |
| F | 138(24.7%) | 188(37.6) |
| Age |  |  |
| <60 | 422(75.5%) | 410(82.0%) |
| ≥60 | 137(24.5%) | 90(18.0%) |
| Tumor |  |  |
| T1-2 | 231(41.3%) | n.a. |
| T3-4 | 328(58.7%) | n.a. |
| LN status |  |  |
| Negative | 43(7.7%) | n.a. |
| Positive | 516(92.3%) | n.a. |
| Distant Metastasis |  |  |
| Negative | 492(88%) | n.a. |
| Positive | 67(12%) | n.a. |
| Stage |  |  |
| I-II | 22(3.9%) | n.a. |
| III-IV | 537(96.1%) | n.a. |
| Histology (WHO) |  |  |
| Keratinizing | 12(2.1%) | n.a. |
| Non-Keratinizing | 527(94.3%) | n.a. |
| Unknown | 20(3.6%) | n.a. |

Abbreviations: LN, lymph node; n.a, not applicable.
