# Supplementary material for: Prognostic Value of Clinical Biochemistry-Based Indexes in Nasopharyngeal Carcinoma
Source: Front Oncol. 2020 Mar 6;10:146. doi: 10.3389/fonc.2020.00146 (PMC7068812; doi:10.3389/fonc.2020.00146)
Supplement: Table S2 — Therapeutic regimens of NPC patients. [file Table_2.docx]

Table S2 Therapeutic regimens of NPC patients

| Therapeutic regimen | survival analysis(n=255) | side-effect analysis(n=500) |
| --- | --- | --- |
| Therapy (on admission ) |  |  |
| Untreated | 8(3.14%) | 52 (10.4%) |
| Chemotherapy alone | 15(5.88%) | 27 (5.4%) |
| Chem-radiotherapy | 209(81.96%) | 398 (79.6%) |
| Radiotherapy alone | 23（9.02%） | 23 (4.6%) |
| Therapy (out of hospital) |  |  |
| Untreated | 0(0%) | 0(0%) |
| Chemotherapy alone | 15(5.88%) | 0(0%) |
| Chem-radiotherapy | 216(84.71%) | 459(91.8%) |
| Radiotherapy alone | 24(9.41%) | 41(8.2%) |
| Radiotherapy therapy |  |  |
| IMRT | 135(56.25%) | 310(62%) |
| TOMO | 39(16.25%) | 54(10.8%) |
| Unknown | 66(27.5%) | 136(27.2%) |
